# Study of bacterial interactions in reconstituted hydrocarbon-degrading bacterial consortia from a local collection, for the bioremediation of weathered oily-soils

**DOI:** 10.1016/j.btre.2021.e00598

**Published:** 2021-02-10

**Authors:** Shaikha Y. Alsayegh, Mohammad A. Al-Ghouti, Nabil Zouari

**Affiliations:** Department of Biological and Environmental Sciences, College of Arts and Sciences, Qatar University. PO. Box 2713, Doha, Qatar

**Keywords:** Bioremediation, Weathered hydrocarbons, *Bacillus sorensis*, *Bacillus cereus*, *pseudomonas stutzeri*, symbiosis

## Abstract

To enhance the process of bacterial remediation of weathered hydrocarbons, the area of Dukhan, Qatar, was considered as a model for weathering processes. Self-purification by indigenous hydrocarbon-degrading bacteria showed low performance. Biostimulation/seeding using one or another of the indigenous bacteria improved the performance. Symbiosis between three strains dominating the soil; *Bacillus sorensis* D11, *Bacillus cereus* D12, and *Pseudomonas stutzeri* D13, was highly performant for removal of total petroleum hydrocarbons in the weathered soil. D11, the most sensitive, showed the highest performance when mixed with D12 or D13. D12, less performant than D11, was more active on diesel range organics (DRO: C10-C28), similar to D11. D13 showed a metabolic behavior close to commensal and co-metabolic ones. It was more active on hydrocarbons above C29. Combination of the three strains conducted to the removal of at least 80% of C10-C35 organics in the extract at concentrations of 31.1 mg/g TPH-DRO.

## Highlights

• Reconstituted hydrocarbon-degrading bacteria consortia from the weathered sites interact positively and negatively in bioremediation processes.
• Bioaugmentation using endogenous bacteria should be based on selection of the appropriate strains, which can co-growth with inhibition.
• The reconstituted hydrocarbon-degrading bacteria consortia lead to reduction of the lag phases and increase of TPH removal at 10 times soil TPH concentrations.

## 1 Introduction

Through the process of extraction and handling of large volumes of petroleum, some spillages and leakages can occur, as well as disposal of huge petroleum extraction wastes [1]. They represent a large source of pollution, causing harmful effects on living organisms. Environmental pollution by oil components is a great concern due to the hundreds of thousands of hydrocarbons and inorganic molecules that are highly toxic at low concentrations [2]. When oil spills occur, large quantities of oil are discharged into the environment. Similarly, never stop the continuous disposal of oil industry wastes can be just reduced. Another additional issue with oil pollution is the weathering processes, which alter the properties and chemical structure of spilled oil. Discharge of oil in the environment makes it subject to continuous weathering processes from the beginning, which contributes to the instability, recalcitrance to biodegradation, and higher toxicity potentials [3]. Oil weathering is a progressive process, which is made up of complex biological, physical, and chemical processes. The rate of oil remediation highly depends on the type of oil as well as the chemical and physical properties of the oil [4]. The weather conditions, including wind speed, waves, and temperatures differentiate the process of weathering [5]. In this case, the oil-weathering process depends on the location and conditions, to which the oil spill is exposed. Gustitus et al. [6] determined the relationship between the weathering of oil and its effects on oil-mineral aggregates. They showed the increase of asphaltene and polar components with weathering, which is affected, in turn, by a decrease in viscosity. All these considerations could explain the metabolic changes of hydrocarbon-degrading bacteria living in harsh conditions and soils. Al Disi et al. [7] isolated and screened bacterial strains exposed to harsh conditions from Qatari soils, characterized by strong weathering processes. They confirmed that extreme weather and harsh conditions prolong adaptation periods for bacterial growth. Qatari soils polluted with oil represent an appropriate matter to study the oil weathering and its interaction with soil components, diversity of bacteria, and their metabolisms and bacterial interactions in bioremediation processes. In Qatar, the soil is exposed to high evaporation rates, elevated temperatures, UV exposure, and salinity. This means that the oil contaminants have minimal dilution, reside in the soil for longer periods, and have slower dispersion rates [8].

Remediation is the removal, degradation, or conversion of contaminants to rather less harmful or harmless pollutants or substances. Bioremediation hence seeks to use naturally occurring biomaterials and living organisms, especially microorganisms. Bacteria are the major microorganisms in the ecological and biodegradation process, especially in the oil-contaminated soils. Peng et al. [9] and AlKaabi et al. [1] evidenced that the composition of the organic matter in the areas determined the diversity of existing bacteria. Hydrocarbon-degrading bacteria are able to adapt to the nutrient composition, weather conditions, and time of exposure, therefore, diverse and dynamic communities of bacteria can exist in different polluted sites [1,1]. This fact outlines many important guidelines for bacterial bioremediation of soil pollution by petroleum contaminants. Some of hydrocarbon-degrading bacteria include *Acinetobacter* sp., *Streptococcus* sp., *Roseomonas* sp., *Sphingobacterium* sp., *Yokenella* sp., *Bacillus* sp. *Capnocy-tophaga* sp., *Alcaligenes* sp., and *Corynebacterium* sp. [[11], [12], [13]]. These bacteria are naturally found in their habitats and are partially responsible for hydrocarbon degradation in the environment. Some other organisms such as fungi have been proven to degrade hydrocarbons in the soil but up to a limited level of degradation and taking longer periods of action [14]. The most primary ways to remove oil products from fresh and weathered solids include the biodegradation of the existing hydrocarbons by microorganisms that exist in their natural habitats [15,16]. Adding nutrients for an appropriate carbon/nitrogen/phosphorous ratio and surfactants to enhance bioavailability represent the biostimulation alternative in the objective of a better activity of the existing microbial communities. Besides, bioremediation can be achieved through various ways of introducing bacteria into the polluted areas, with the condition that the formed bacterial consortia be able to use the hydrocarbons as substrate and adapt to the external conditions that surround their habitat [17,18]. Bioaugmentation or commonly known as seeding is referred to the addition of bacterial consortia, that are highly concentrated with selected populations (which may be consortia or single strains), to the contaminated area containing the recalcitrant elements [19]. This process tends to take a natural approach in dealing with microbial ecology, especially their interactions and co-existing. Here, some of the parameters that need to be taken into consideration during the introduction of such bacterial mixtures in the soils include water, oxygen, nitrogen, and phosphorus [20].

The bioaugmentation process is more commonly used in areas that have insufficient consortia of bacteria. In the case of weathered hydrocarbons, seeding is necessary to introduce new metabolic pathways, which can deal with the existing substrates. However, co-metabolism and commensalism, well-established concepts in hydrocarbon-degrading bacteria, also depend on the acceptors of electors employed by each individual bacterial strain, chemical conversion, and intermediates, the toxicity of the intermediates, and their role as substrates for other members of the community, among others [21]. This makes seeding very sensitive and may be the source of failure of most of the bioaugmentation approaches employed in the arid areas or those areas characterized by harsh conditions, like Qatar. Consequently, it is now well established that endogenous bacteria are more appropriate to remediate the corresponding polluted soils than exogenous ones. Alkaabi et al. [1,12] and Oualha et al. [13] demonstrated that bacteria from the site of dumping petroleum wastes in Qatar are highly adapted to the weathered hydrocarbons as showed in the site and that biostimulation alone is not efficient if applied solely. When combined with bioaugmentation using the indigenous bacterial strains, better performances were registered. However, the complete and the fast removal were not achieved. This was attributed to the interaction between the bacterial communities. The aim of this study is to evaluate the interactions between three bacterial strains that were previously isolated from contaminated weathered petroleum soil in the oily wastes dumping site in Qatar. These bacterial strains are identified as *Bacillus sorensis* D11*, Bacillus cereus* D12 and *Pseudomonas stutzeri* D13 by using advanced molecular techniques. Their high activity was previously evaluated in the soil with low efficiency. Many complex interactions between them were suspected in the soil. Which one or which combination is appropriate for weathered soil is still the unanswered question. The answers would fill a gap in our knowledge of the potential positive and negative interactions between bacteria growing in weathered oil. Here, the weathered hydrocarbons were extracted from soil and the questions related to these interactions are investigated in liquid MSM medium. This simulated situation in a better controlled growth conditions will define the behavior of individual and reconstituted consortia of endogenous bacteria. Growth in terms of colony-forming units, removal of the total petroleum hydrocarbons (TPH) including the diesel range organics (TPH-DRO) and the oil range organics (TPH-ORO), and changes in the extract composition studied by Fourier transform infrared (FTIR) were the basis of this work. This is important to consider in the selection and seeding of the appropriate bacterial strain.

## 2 Material and methods

### 2.1 Soil samples

Composite soil samples were collected from 10 locations, each distanced 10 m from the other, in the site of dumping liquid and solid oily wastes in the industrial area of Dukhan, in Qatar. It is a highly controlled area in which ground and surface pollution is prevented. When it is completely full, each basin of the site is left to open air for self-remediation. The selected basin was drying for 3 years before sampling. Alkaabi et al., [12] showed that a small oil fraction, which is relatively unweathered, was found inside heavily weathered oil that solidiﬁed and thus prevented further degradation of the interior oil. Sampling was performed by using sterile tools for collecting polluted soil. The samples were preserved into a sterile polypropylene container and store a +4 °C until use.

### 2.2 Extraction of total petroleum hydrocarbons (TPH)

The soil was first dried during 48 h in an oven set at 50 °C without ventilation. Then, it was ground and sieved with 2 mm sieve to homogeneity. Total petroleum hydrocarbons (TPH) were extracted from homogenized soil, placed into extraction vials after the addition of 1 g of diatomaceous in each vial of 1 g soil. Then, dried alumina (20 g) was added to each vial, tightly sealed, and placed in the ASE DINOX SE 500 evaporator for extraction, using methylene chloride/acetone (1:1, v/v). An accelerated solvent extractor machine was used with a pre-programmed extraction cycle. Complete extraction took 30 min at 175 °C. All the extracts were harvested and mixed, evaporated under pure nitrogen, and ﬁltered and washed using solid-phase extraction (SPE) cartridges (silica column). The final volume of extracts was 30 mL (from 30 g soil), used for TPH analysis, and as a substrate in the cultural media.

### 2.3 Analysis of total petroleum hydrocarbons (TPH)

The TPH was analyzed in the extracts and the cultural media (after extraction with methylene chloride/acetone (1:1, v/v) by Gas Chromatography – Flame Ionization Detector (GC-FID) as detailed and reported by Alkaabi et al. [12]. TPH including the diesel range organics (TPH-DRO) and the oil range organics (TPH-ORO) were determined according to AlKaabi et al. [12] and Christensen et al. [22,23] and their removal was calculated accordingly.

### 2.4 Fourier transform infrared (FTIR) analysis of the culture media

The initial soil sample, the extracts, and the culture media (before and after bacterial growth) were analyzed by FTIR. First, all samples were evaporated and dried in an oven set at 50 °C without ventilation, until a stable weight of the dried matter. The FTIR Perkin Elmer 400 FT-IR/FT-NIR spectrometer was used with a pressure on the samples ranging from 30 to 50 psi. The spectra were recorded in the range of 400–4000 cm^−1^. The method was described by Alkaabi et al. [12] and Oualha et al. [13].

### 2.5 Media, bacterial strains and culture conditions

Three bacterial strains named *Bacillus sorensis* D11, *Bacillus cereus* D12, and *Pseudomonas stutzeri* D13 were used in this study. They were previously isolated from the same soil used for the extraction of hydrocarbons in this study and identified by ribotyping [1,13]. Cultures were performed in MSM medium as previously optimized and published by Attar et al. [10]. The initial pH was 7.2, as that of the used soils. The C/N/P was adjusted to 100/10/1 using ammonium nitrate as a nitrogen source, and a mixture of KH_2_PO_4_/Na_2_HPO_4_ and as phosphorus source Attar et al. [10]. All the experiments were performed in a shaker set at 37 °C and 200 rpm. First, each bacterial strain was cultured in a sterile falcon tube containing 20 mL LB medium, inoculated with two colonies of an overnight culture on solid LB plates. After 24 h incubation at 37 °C and 200 rpm, the culture broths were centrifuged and the pellets were washed twice with MSM medium and suspended in 5 mL MSM to serve as inoculum. Then, the optical density (OD) of each inoculum was measured at 600 nm. The volume of the inoculum introduced in the culture was 0.15 at the inoculation time (initial time of the cultures). It corresponded to 0.357 + 0.0310^7^ cfu/mL, 0.478 + 0.03 10^7^ cfu/mL and 0.443 + 0.03 10^7^ cfu/mL for D11, D12 and D13, respectively. By mixing two strains or the three strains, the volume of each inoculum was divided by two or three times, respectively to ensure similar cultural conditions to all cultures regarding the inoculum percentage. During incubation, 500 μL were taken out from the cultures, at sterile conditions, and the corresponding cfu were determined.

### 2.6 Determination of the colony-forming units (cfu)

**Fig. 1:**
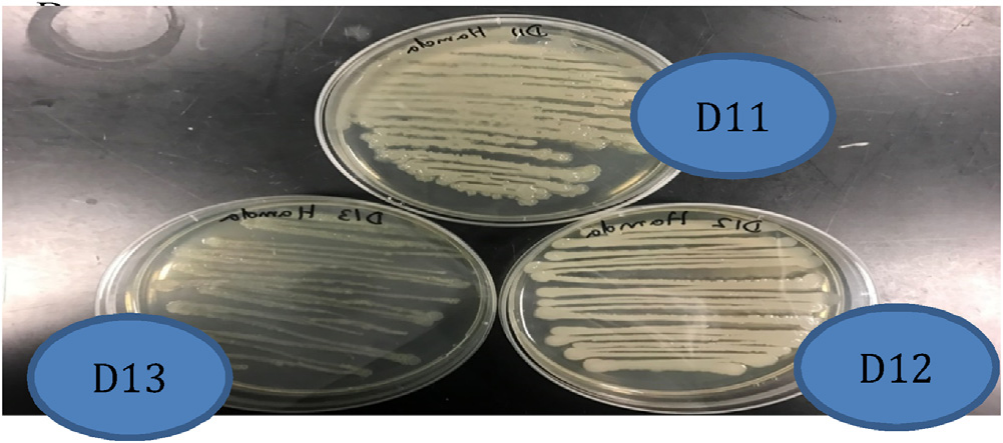
Distinctive colony characteristics of the strains D11 *Bacillus sorensis*, D12 *Bacillus cereus,* and D13 *Pseudomonas stutzeri* on solid LB medium.

To determine the colony forming units (cfu) in the cultural media, serial dilutions were prepared from each culture sample and 100 μL from each dilution were spread on LB plates, incubated 48 h at 37 °C, the colonies counted and the corresponding cfu calculated [13]. Regarding the mixtures of the strains, the cfu corresponding to each strain was determined by counting their respective colonies. The growth of these bacteria on solid LB media showed different shapes and characteristics, which may be easily identified as shown in Fig. 1. The growth of D11 is associated with the overproduction of surfactants, making highly shining colonies. The colonies of D12 are white with clear surfactants surrounding the colony. Colonies of D13 are strongly white, much smaller than those of the two other strains, slightly yellowy and less producing surfactants (not shining).

### 2.7 Results and discussion

To study the behavior of each bacterial strain and their mixtures in liquid MSM medium with weathered hydrocarbons, the extraction of TPH was performed from Dukhan soils according to the modified Accelerated Solvent Extraction (ASE, U.S. EPA Methods 3540). This method allows extraction of hydrocarbons referred to as GRO (gasoline range organics in the C_6_-C_10_ range), DRO (diesel range organics in the C_10_-C_28_ range) extended to ORO (Oil Range Organics) up to C_35_. TPH is a large family of compounds (which may be more than several hundred chemicals) that originated from crude oil. They are a mixture of hydrocarbons, made mainly from hydrogen and carbon. They serve as a carbon source for hydrocarbon-degrading microorganisms. The TPH is divided into groups acting alike in water and soil. However, they are used as a parameter of pollution by oil, and their removal is an indicator of a successful remediation strategy. The biodegradability of classes of TPH organics is variable. The most susceptible to biodegradation are *n*-alkanes and isoprenoid aliphatic alkanes [24]. Monoaromatic steroids, diasteranes, 25-norhopanes, C_21_-C_22_ steranes, tricyclic terpanes, oleanane, gammacerane and diahopanes are the most resistant [25]. Regarding the extracted TPH from the weathered soil of the Dukhan dumping site in Qatar, the analysis provided a concentration of 93.3 mg/mL TPH-DRO (and diesel range organics) and 594 mg/mL TPH-ORO (Oil Range Organics). This means that, initially, the soils contained 3.11 mg/g TPH-DRO and 19.8 mg/g TPH-ORO. It is then clear that the fraction C_29_-C_35_ hydrocarbons represent 6.4 folds of the DRO fraction (C_10_-C_28_ range). This is expected since the DRO hydrocarbons are more biodegradable than the C_29_-C_35_ hydrocarbons, then mostly removed during the weathering process. Since TPH-organics represent the only carbon source in the medium, their removal or transformation should result in the growth of hydrocarbon-degrading bacteria. The growth and viability of the bacterial cells should provide an appropriate indicator of the ability of the bacterium to use hydrocarbons but also tolerate the toxicity exhibited by the hydrocarbons or their derivatives through the metabolic pathways. In order to evaluate the impact of the growth of each strain on TPH removal, the removed fractions of DRO and ORO of TPH in each culture was calculated. Besides, the ratio ORO/DRO of the residual fractions was calculated, knowing that the initial ORO/DRO was almost 6.4. It is expected that the increase of the ORO/DRO ratio in the cultures should correspond to the removal of more hydrocarbons in the range of DRO than the C_29_-C_35_ ones, as extended to ORO. The inverse is correct.

### 2.8 Evaluation and comparison of the growth of the seeded bacteria at low concentration of TPH-hydrocarbons

**Fig. 2:**
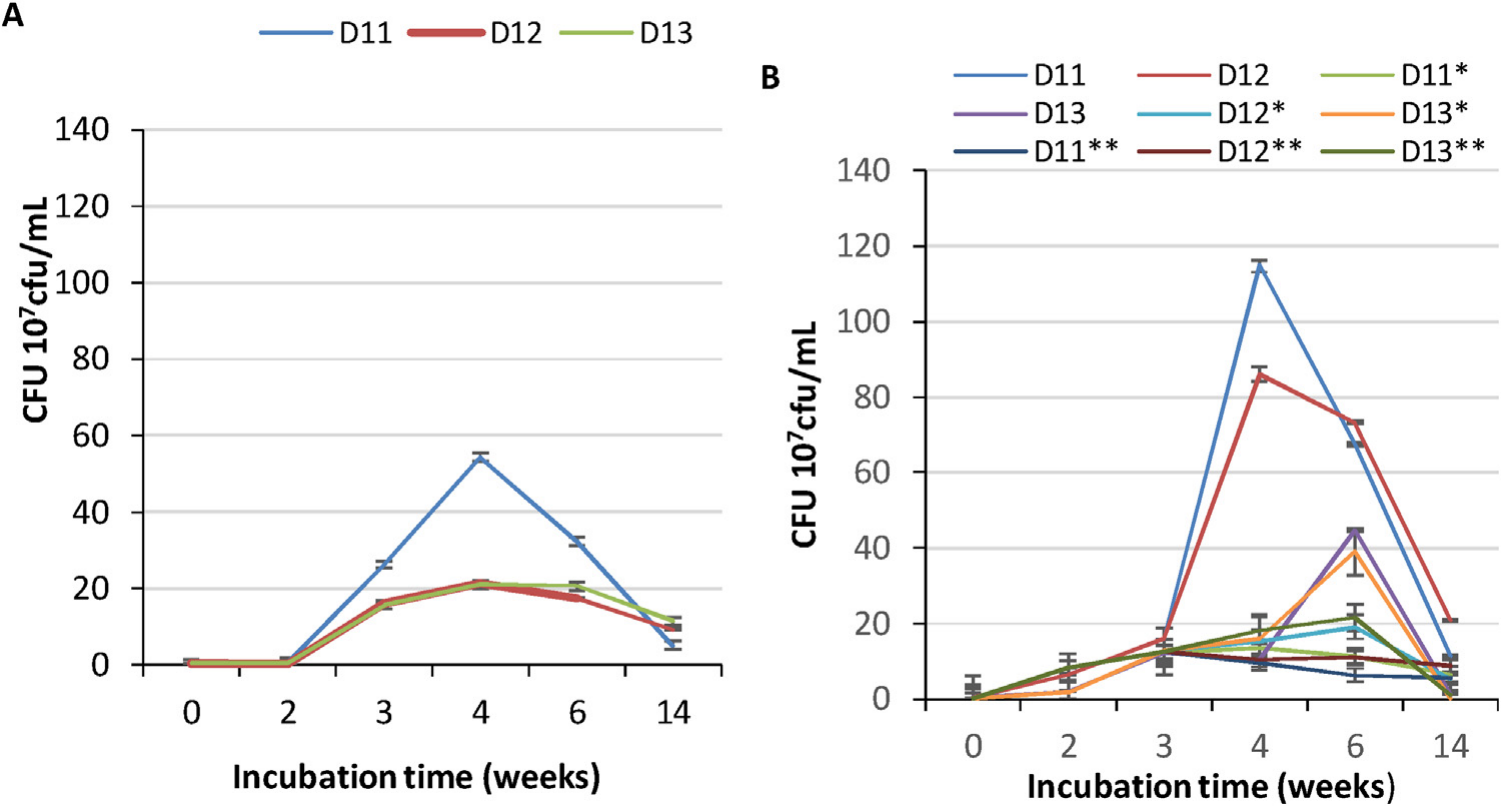
Evaluation of growth in 0.311 mg TPH-DRO corresponding to 198 mg/mL TPH-ORO. Cells counts are presented as 10^7^ cfu/mL. A: Cultures inoculated with separate bacterial strains. B: cultures inoculated with bacterial mixtures: D11 in D11/D12; D11* in D11/D13; D11** in D11+D12+D13; D12 in D12/D11; D12* in D12/D13; D12** in D12/D11/D13; D13 in D13/D11 and D13* in D13 + D12.

The growth of the three bacterial strains was evaluated at low TPH concentrations, to avoid potential toxicity effects on each of the strains. The culture medium was supplemented with the weathered TPH extract, ensuring concentrations of 0.311 mg/mL TPH-DRO in 1.98 mg/mL TPH-ORO. Since the shape, color, and size of the colonies formed by each isolate were distinctive (Fig. 1) at most of the studied incubation periods, it was possible and feasible to evaluate the growth of each of them at some incubation times. These concentrations correspond to 10% of that in the soil. The growth of each strain, cultured separately, was followed during the incubation periods by counting the cfu. The results are shown in Fig. 2A. The bacterial strains D11, D12, and D13 started growing slowly during the two first weeks (Fig. 2A). D11 doubled the biomass, D12 showed a decrease of its biomass and D13 maintained almost the initial biomass. This represents long lag phases of growth. However, there was a clear growth observed in the first two weeks for the four combined sets of the bacterial strains D11/D12, D11/D13, D12/D13, and D11/D12/D13 (Fig. 2B). Indeed, with D11/D12 and D11/D12/D13 combinations, the biomass reached 13 10^7^ cfu/mL and 25 10^7^ cfu/mL, respectively, while it does not exceed 0.77 × 10^7^ cfu/mL at the most for D11. This observation proves that the mixed bacterial strains could reduce the lag phase during the first weeks. Since the bacterial cells are introduced into the medium whose substrates are hydrocarbons, they have to adapt to the conditions of the culture. With all the three strains, the exponential phase occurred in the period ranging from week 2 to week 3 to reach 37.2 × 10^7^ cfu/mL with the consortium made with the three strains and 24.4 × 10^7^ cfu/mL to 32 × 10^7^ cfu/mL for the other combinations. During this phase, the bacterial cells experience the most rapid growth possible under the conditions of the cultures. The count of cells increases at the rate proportional to the number of cells present at any given time. When the three strains were incubated separately, D11 (*Bacillus sorensis*) showed the highest growth rate of all the three strains. On the other hand, D13 (*Pseudomonas stutzeri*) experienced the least rate of growth. This difference in the rate of growth could be attributed to the difference in concentrations among the three strains. In this case, D11 could have the highest concentration of the three. The stationary phase possibly took place in week 4 by all the three strains incubated separately. The results show that the peak of growth took place in the fourth week. The stationary phase is characterized by the lack of clear counts increase. One possible explanation for the lack of growth in this phase is the depletion of the carbon and energy sources from the hydrocarbons available in the medium and which are susceptible to be consumed by the bacterial cells. In addition, this may be explained by the buildup of waste products in the culture, exhibiting an additional possible toxic effect. The death phase took place from the end of week 4 as shown by the decline in cell counts in all the three separately incubated strains. This phase is characterized by the net loss of viable cells. This phase occurs due to the complete depletion of nutrient source susceptible to maintain the bacterial cells. For the combined sets, various observations were made. First, for D11/D12 combination, the growth of both strains peaked in week 4, hence the stationary phase could have started in week 4 for both strains. When D11 and D13 were incubated together, D13 exhibited the highest growth in week 6 while D11 peaked in week 4. These results show that 0.311 mg/mL TPH-DRO in the1.98 mg/mL TPH-ORO do not provide enough readily assimilable substrates and cause a long adaptation period of the three bacteria, needed to start consuming the available substrates. D11 is more efficient in such activity, but also more sensitive to exhaustion of substrates or the toxic effect of intermediates or end-products. A combination of 2 or 3 strains, allowed the decrease of the lag phase, since cell counts jumped to almost 4 × 10^7^ cfu/mL with D11/D13 and D12/D13 combinations, while D11/D12 allowed production of 13 × 10^7^ cfu/mL. Interestingly, the combination of the three strains lead to a production of 25 × 10^7^ cfu/mL. By week 4, with the D11/D12 combination, the highest counts of D11 (double than that in individual culture) and of D12 (4 times than that in individual culture) were obtained. These results show a positive interaction between the two strains, with a higher benefit for D12 than D11. The decrease of biomass was less rapid than that observed in separate cultures. In all other combinations, the stationary phase followed by the continuous and slight decrease in the biomass of each strain was established starting the 3rd week. It is then clear that the common positive effect for all the combinations was the decrease of the lag (adaptation) phase. An interesting combination of D11/D12 may be registered for further studies.

Table 1 shows the performance of the strains in the removal of TPH-DRO and TPH-ORO with an initial concentration of 0.311 mg/mL TPH -DRO in 1.98 mg/mL TPH-ORO. The strain D11 can achieve 38% removal of TPH-DRO and 27% of TPH-ORO after 14 weeks incubation. This means that D11 is more active on the TPH-DRO range, leading to an increase in the ratio ORO/DRO to 7.5 compared to the initial one of 6.4. D12 was less performant to remove TPH than D11. Its corresponding ORO/DRO ratio was also increased to 7.5. D13 was much less performant that D11 and D12, although showing capabilities to remove TPH molecules, without no preference to DRO (the ORO/DRO ratio was maintained close to 6.4. Interestingly, the combination D11/D12 provided 79% TPH removal and the combination D11/D12/D13 allowed removal of 87% of TPH. The activity was similar to TPH-DRO and TPH-ORO with both combinations. However, the combination D11/D13 was able to improve slightly TPH removal, but with the increase of the ORO/DRO ratio. It seems that the co-metabolic activities of D11 and D12 would be interesting. D13, which was more active on C_29_-C_35_ molecules than C_10_-C_28_ can only improve the TPH removal in the combinations D11/D12 or D11/D12/D13. The symbiosis of D13 to D11 and D13 seems to be closer to a commensal one more than a mutualistic one.

**Table 1:** Removal of TPH -DRO and TPH-ORO with bacteria at initial concentrations of 0.311 mg/mL TPH -DRO and 1.98 mg/mL TPH-ORO.

TPH Removal (%)											
Strains	Week 2		Week 3		Week 4		Week 6		Week 14		
	DRO	ORO	DRO	ORO	DRO	ORO	DRO	ORO	DRO	ORO	Residual ORO/DRO
D11	11+1	4+1	15+1	6+1	18+2	11+1	29+2	19+2	38+2	27+2	7.5
D12	7+1	2+1	10+1	4+1	13+2	8+1	18+2	11+2	23+2	16+2	7.61
D13	6+1	2+1	8+1	5+1	11+2	8+1	15+2	9+2	19+2	17+2	6.5
D11+D12	33+1	14+1	42+1	22+1	58+2	31+1	69+2	47+2	78+2	79+2	6.3
D11+D13	17+1	9+1	25+1	16+1	31+2	21+1	39+2	29+2	48+2	37+2	7.7
D12+D13	19+1	11+1	23+1	17+1	29+2	22+1	41+2	31+2	53+2	44+2	7.6
D11+D12+D13	37+1	24+1	52+1	31+1	68+2	47+1	81+2	69+2	86+2	87+2	6.3

### 2.9 Evaluation and comparison of the growth of the seeded bacteria at high TPH hydrocarbons concentrations

**Fig. 3:**
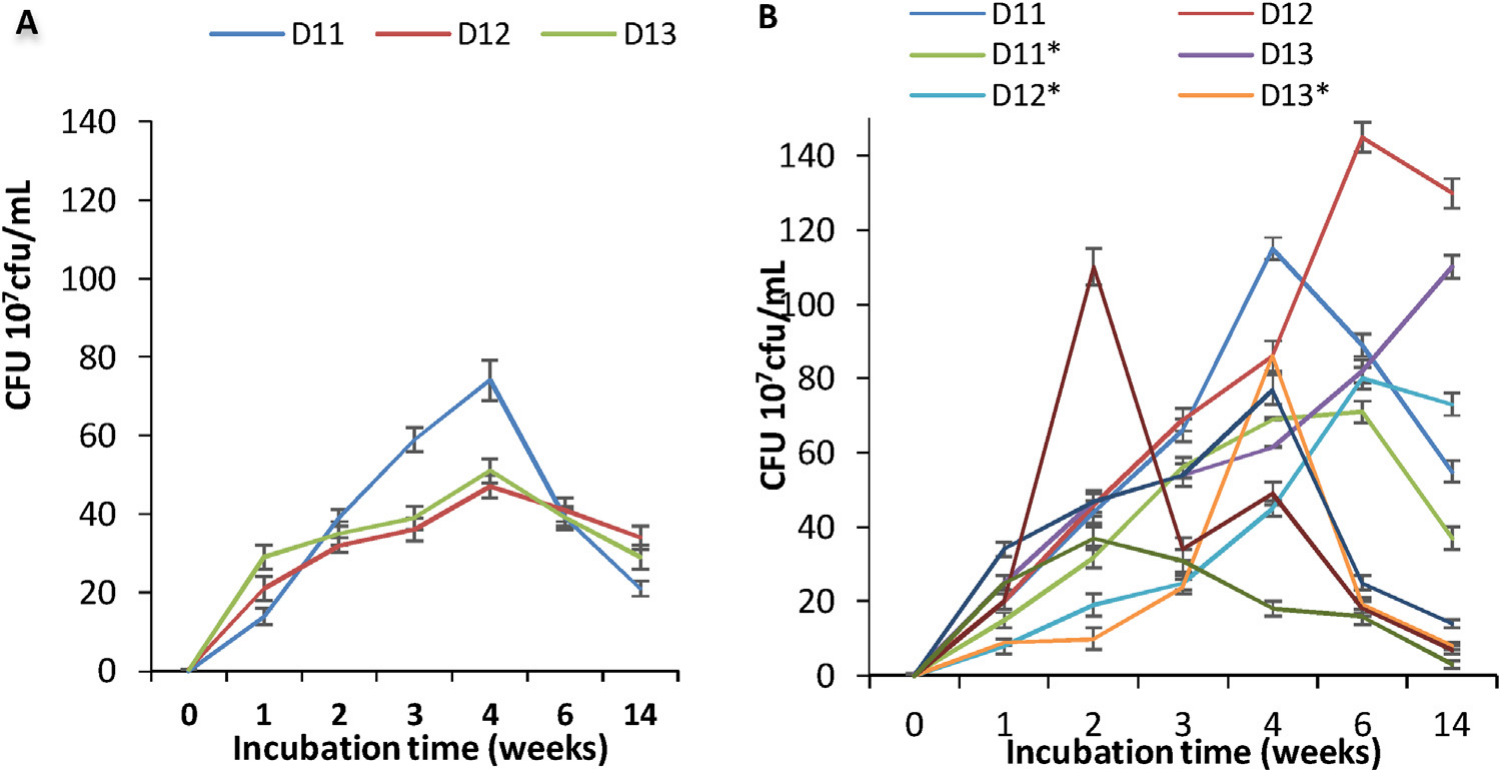
Evaluation of growth in 1.55 mg/mL TPH-DRO corresponding to 9.9 mg/mL TPH-ORO. Cells counts are presented as 10^7^cfu/mL. A: Cultures inoculated with separate bacterial strains. B: cultures inoculated with bacterial mixtures: D11 in D11/D12; D11* in D11/D13; D11** in D11+D12+D13; D12 inD12/D11; D12* in D12/D13; D12** in D12/D11/D13; D13 in D13/D11 and D13* in D13 + D12.

**Fig. 4:**
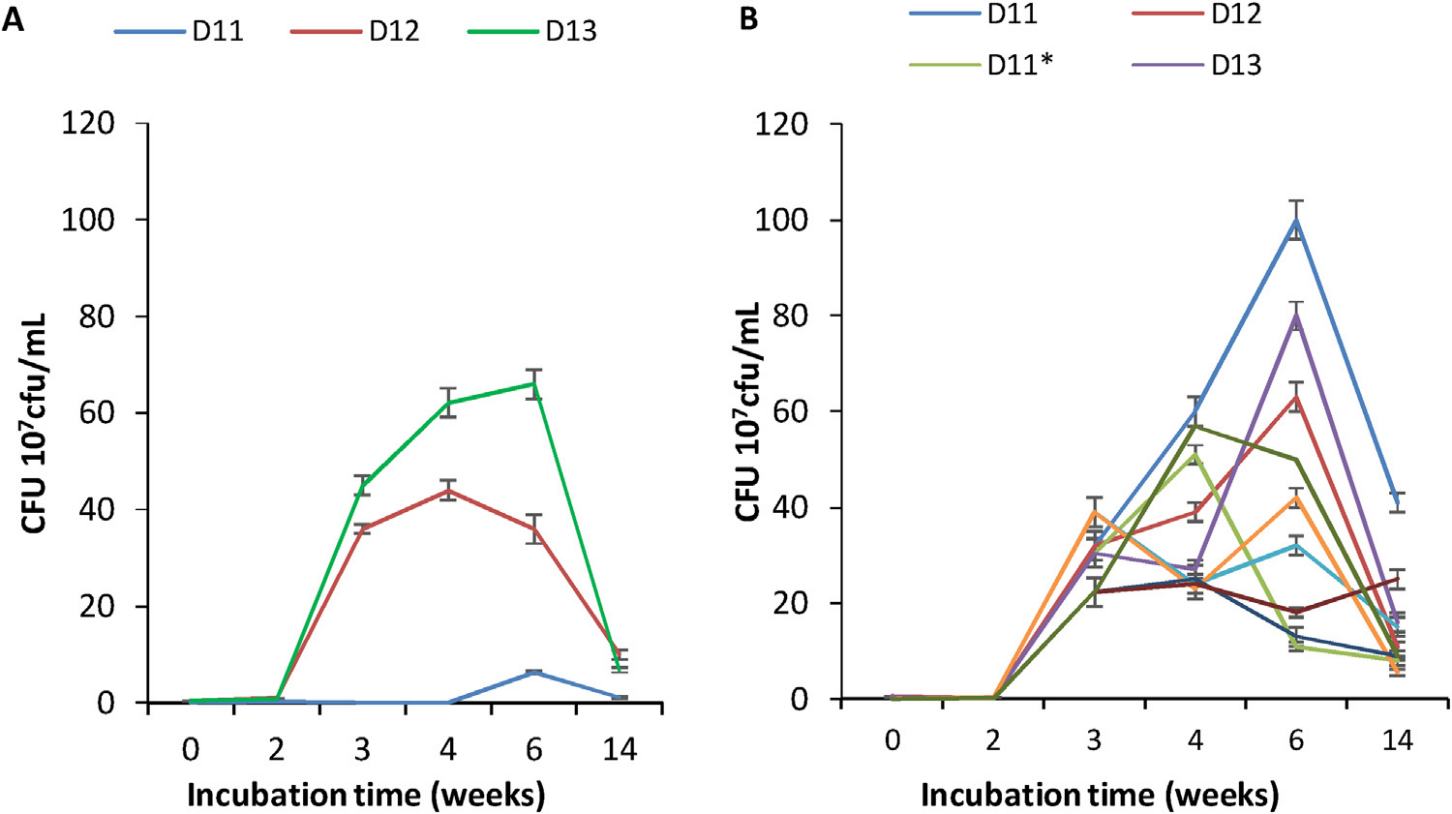
Evaluation of growth in 3.11 mg/mL TPH-DRO corresponding to 19.8 mg/mL TPH-ORO. Cells counts are presented as 10^7^cfu/mL. A: Cultures inoculated with separate bacterial strains. B: cultures inoculated with bacterial mixtures: D11 in D11/D12; D11* in D11/D13; D11** in D11+D12+D13; D12 inD12/D11; D12* in D12/D13; D12** in D12/D11/D13; D13 in D13/D11 and D13* in D13 + D12.

**Fig. 5:**
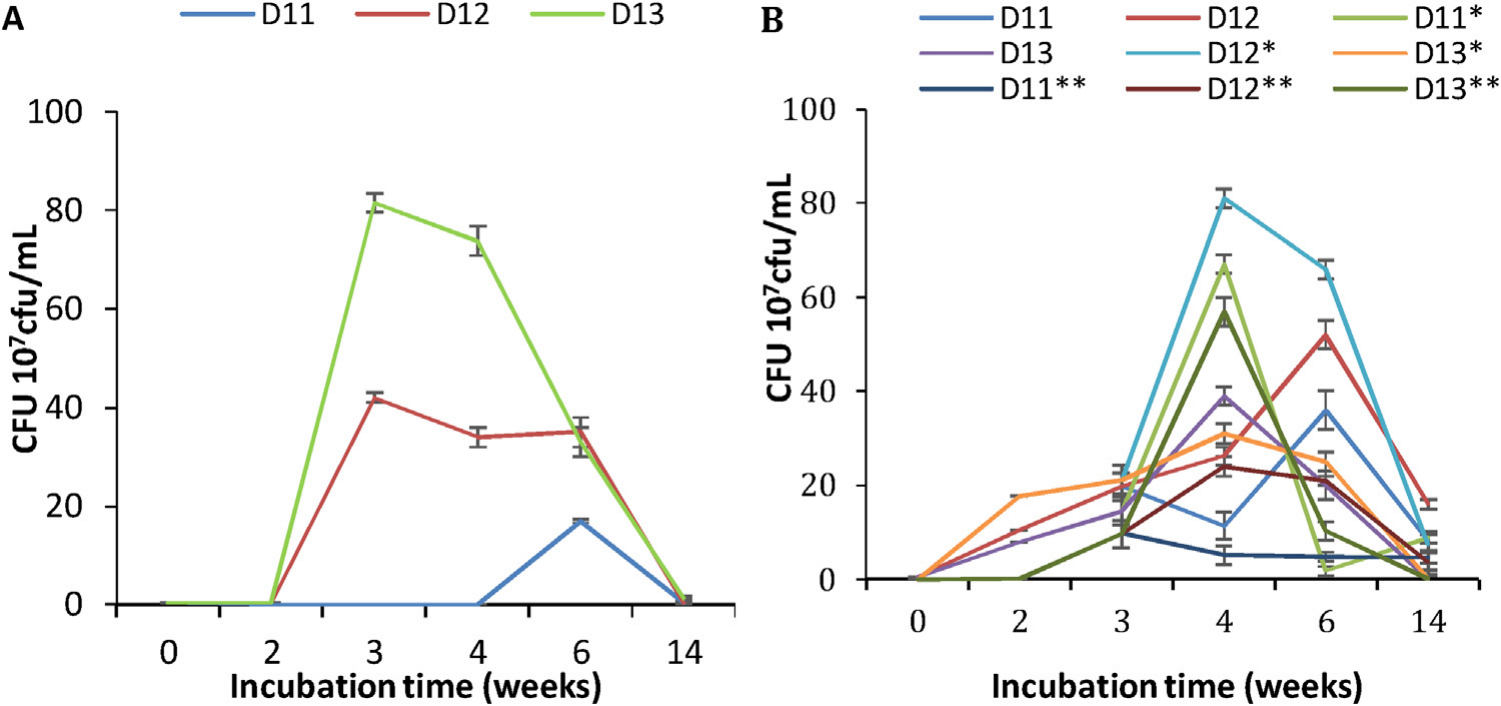
Evaluation of growth in 31.1 mg/mL TPH-DRO corresponding to 198 mg/mL TPH-ORO. Cells counts are presented as 10^7^ cfu/mL. A: Cultures inoculated with separate bacterial strains. B: cultures inoculated with bacterial mixtures: D11 in D11/D12; D11* in D11/D13; D11** in D11+D12+D13; D12 inD12/D11; D12* in D12/D13; D12** in D12/D11/D13; D13 in D13/D11 and D13* in D13 + D12.

The effect of higher concentrations of hydrocarbons, corresponding to five times of that used in the first study, was investigated (Fig. 3). The concentration of hydrocarbons was 1.55 mg/mL TPH-DRO in 9.9 mg/mL TPH-ORO. After one-week incubation, the cell counts showed a pronounced increase, showing a short lag phase. Compared to the lower TPH concentrations results (Fig. 2), the lag phase with all the combinations was much reduced and the bacterial growth reached the exponential phase from week 3 to week 4 with higher cell counts than that with the lower TPH concentration. The stationary and death phases were delayed, except for D11, which showed a high sensitivity to 1.55 mg/L TPH-DRO. The growth of the isolates and their combinations at such concentration of TPH accelerated the adaptation of the bacterial cells and informed about the starting effect of toxicity of some components of the extract to D11 and D12 as pure cultures (Fig. 3A). When these two bacterial strains were co-cultured in all the combinations, they exhibited more resistance to such toxicity (Fig. 3B). However, in week 6 and 14, the growth increased in the combinations compared to the lower TPH concentration. This was not the case in pure cultures. As hydrocarbons concentrations increased to 10 times providing 3.1 mg/mL TPH-DRO in 19.8 mg/mL TPH-ORO, no growth in week 1 followed by moderate growth on week 2 was noticed (Fig. 4). However, growth began to decrease from week 3 until week 14 for all strains in separate and mixed cultures. The results of Fig. 5, obtained with a high concentration of TPH (31.1 mg/mL TPH-DRO in 198 mg/mL TPH-ORO, representing 100 times higher than those of Fig. 2), show that the lag phase was interestingly reduced to less than two weeks in the combinations D11/D12, D11/D13, and D12/D13 only. However, high toxicity was exhibited on all the strains in these co-cultures starting week 4. It is obviously clear that the behavior of the separate isolates or their combinations is different at each hydrocarbons concentration. D11 and D12 are the most sensitive to toxicity.

To confirm the performance of all strains and the symbiosis in their combinations, 10-times and 100 times concentrated TPH were used (Fig. 4, Fig. 5) similarly to the 1-time and 5-times TPH concentrations (Fig. 2, Fig. 3). As TPH concentrations increased to10 times, providing 3.11 mg/mL TPH-DRO in 19.8 mg/mL TPH-ORO, the results of Fig. 4 show that there was no growth in the week 1, followed by moderate growth on week 2. However, growth began to decrease from week 3 until week 14 of all-separate and mixed strains. The strain D11 was highly sensitive to the toxicity exhibited by the TPH at such concentration. However, in the combinations of strains, D11 showed a better tolerance to TPH toxicity. The same conclusion can be drawn for D12 and D13. Results of Fig. 5 were obtained with high concentrations of 31.1 mg/mL TPH-DRO and 198 mg/mL TPH-ORO, compared to the concentrations used in Fig. 2. Interestingly, the lag phase of growth of all strains combined together was reduced to less than one week, after what a high growth was registered. However, starting the week 6, growth decreased in all cultures of separate or mixed strains. This shows that the growth and use of TPH as carbon and energy sources of each separate or combined strains depends on the concentration of each component in the TPH extract. Symbiosis between the bacterial communities seems also to be affected by the concentration of TPH. Indeed, considering the Monod theory, each component that can be used as a substrate has a specific saturation constant Ks for each bacterial strain. This constant determines, in turn, the affinity of the substrate to the bacterium. Knowing the difficulties, which can be encountered by the bacterial metabolism to mineralize totally the substrate, one can expect intermediates of the metabolic activities, which serve in turn as a substrate to other bacteria or metabolized by co-metabolism, using other substrates or intermediates.

Using 1.55 mg/mL TPH-DRO in 9.9 mg/mL TPH-ORO, the removal efficiencies were improved in and all the strains combination, although the combination D11/D12/D13 exhibited similar results (84%) than those with lower TPH concentrations (Table 2). However, D11 and D12 clearly showed much higher activities on C_10_-C_29_ hydrocarbons than C_29_-C_35_, which confirms the results obtained with lower TPH concentrations. D13 which removed similarly both categories of hydrocarbons at low concentration exhibited more activity on the TPH-DRO at the 5 times concentrated TPH in the medium. The combination D11/D12 was similarly active on C_10_-C_29_ hydrocarbons and C_29_-C_35_, maintaining the ORO/DRO stable at 6.4, as shown with lower TPH concentration. The D11/D13 was more interesting in the removal of C_10_-C_29_ hydrocarbons at the 5-times concentrated TPH in the medium. All the combinations showed an interesting removal profile and the commensalism of D13 is still applicable at the studied concentration.

**Table 2:** Removal of TPH -DRO and TPH-ORO with bacteria at initial concentrations of 1.55 mg/mL TPH-DRO corresponding to 9.9 mg/mL TPH-ORO.

Removal (%)											
Strains	Week 2		Week 3		Week 4		Week 6		Week 14		
	DRO	ORO	DRO	ORO	DRO	ORO	DRO	ORO	DRO	ORO	Residual ORO/DRO
D11	18+1	8+1	26+1	11+1	32+2	14+1	37+2	19+2	41+2	22+2	8.4
D12	11+1	3+1	15+1	6+1	18+2	11+1	23+2	14+2	32+2	18+2	7.7
D13	13+1	4+1	17+1	8+1	20+2	12+1	22+2	14+2	28+2	17+2	7.3
D11+D12	23+1	11+1	30+1	19+1	37+2	26+1	43+2	37+2	53+2	54+2	6.2
D11+D13	17+1	13+1	22+1	16+1	29+2	19+1	37+2	22+2	48+2	49+2	6.2
D12+D13	16+1	11+1	20+1	15+1	27+2	20+1	36+2	27+2	42+2	36+2	7.0
D11+D12+D13	41+1	16+1	49+1	28+1	54+2	37+1	63+2	48+2	73+2	74+2	6.1

Regarding TPH removals using 3.11 mg/mL TPH-DRO in 19.8 mg/mL TPH-ORO (Table 3) or 31.1 mg/mL TPH-DRO in 198 mg/mL TPH-ORO (Table 4), it is obviously clear that the removal efficiencies were decreasing with the increase of TPH concentrations, which is expected. However, after 14 weeks incubation, the combinations D11/D12, D11/D13 and D11/D12/D13 exhibited a decrease of ORO/DRO ratios, which means that their synergic metabolic activities were efficient on C_29_-C_35_ that are more difficult to be removed than TPH-DRO (C_10_-C_29_ hydrocarbons). Indeed, at 31.1 mg/mL TPH-DRO in 198 mg/mL TPH-ORO, the consortium of the three bacterial strains exhibited around 70% removal of both of them. The ORO/DRO ratio was significantly decreased down to 5.5, instead of 6.4 at the beginning of the incubation. The symbiosis between the strains was so efficient at such concentrations.

**Table 3:** Removal of TPH -DRO and TPH-ORO with bacteria at initial concentrations of 3.11 mg/mL TPH-DRO corresponding to 19.8 mg/mL TPH-ORO.

Removal (%)											
Strains	Week 2		Week 3		Week 4		Week 6		Week 14		
	DRO	ORO	DRO	ORO	DRO	ORO	DRO	ORO	DRO	ORO	Residual ORO/DRO
D11	13+1	5+1	22+1	10+1	27+2	15+1	34+2	19+2	37+2	20+2	8.1
D12	9+1	4+1	14+1	8+1	19+2	13+1	24+2	17+2	28+2	17+2	7.3
D13	11+1	3+1	15+1	6+1	18+2	12+1	21+2	15+2	26+2	18+2	7.1
D11+D12	25+1	8+1	27+1	13+1	36+2	23+1	44+2	31+2	49+2	51+2	6.1
D11+D13	18+1	7+1	26+1	16+1	30+2	22+1	34+2	28+2	44+2	43+2	6.5
D12+D13	13+1	8+1	18+1	12+1	21+2	18+1	26+2	22+2	32+2	27+2	6.8
D11+D12+D13	33+1	14+1	42+1	22+1	50+2	33+1	59+2	44+2	69+2	73+2	5.5

**Table 4:** Removal of TPH -DRO and TPH-ORO with bacteria at initial concentrations of 31.1 mg/mL TPH-DRO corresponding to 198 mg/mL TPH-ORO.

Removal (%)											
Strains	Week 2		Week 3		Week 4		Week 6		Week 14		
	DRO	ORO	DRO	ORO	DRO	ORO	DRO	ORO	DRO	ORO	Residual ORO/DRO
D11	29+1	12+1	35+1	14+1	38+2	19+1	45+2	29+2	53+2	37+2	8.5
D12	16+1	5+1	21+1	9+1	26+2	16+1	33+2	19+2	41+2	23+2	8.3
D13	20+1	6+1	26+1	11+1	32+2	18+1	38+2	22+2	44+2	25+2	8.6
D11+D12	42+1	16+1	52+1	39+1	57+2	47+1	62+2	58+2	73+2	74+2	6.3
D11+D13	27+1	15+1	39+1	24+1	55+2	39+1	67+2	52+2	72+2	73+2	6.3
D12+D13	12+1	14+1	27+1	20+1	36+2	27+1	48+2	37+2	59+2	49+2	7.9
D11+D12+D13	44+1	18+1	59+1	42+1	64+2	58+1	71+2	69+2	83+2	84+2	6.3

### 2.10 Impact of the bacterial strains on hydrocarbons of TPH in pure cultures and mixed cultures

**Fig. 6:**
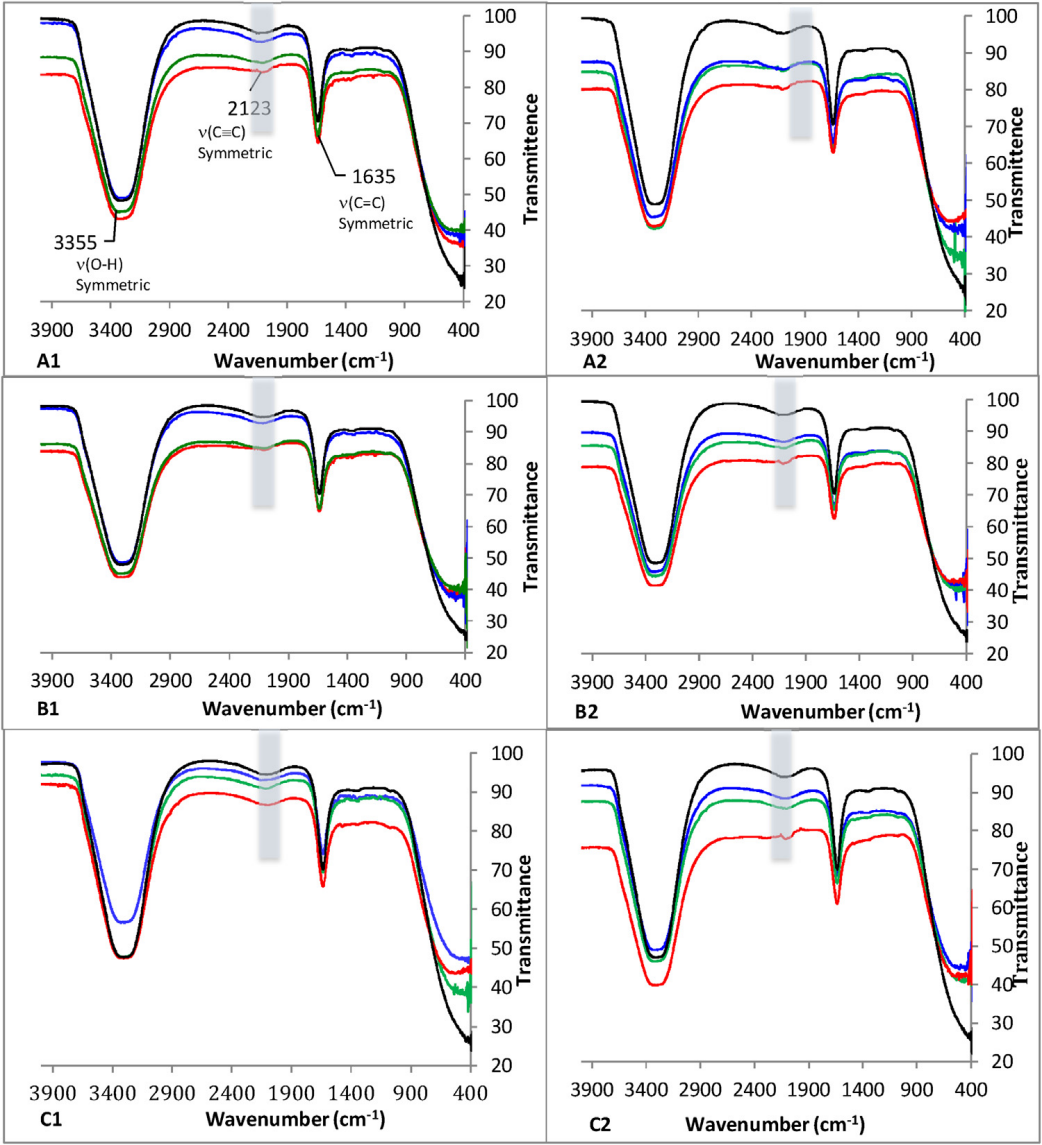
FTIR analyses performed in the cultures performed with : **A1**: D11 in 1.55 mg TPH-DRO corresponding to 9.9 mg/mL TPH-ORO. **A2**: D11 in 31.1 mg TPH-DRO corresponding to 198 mg/mL TPH-ORO. **B1**: D12 in D11 in 1.55 mg TPH-DRO corresponding to 9.9 mg/mL TPH-ORO. **B2**: D12 in 31.1 mg TPH-DRO corresponding to 198 mg/mL TPH-ORO. **C1**: D13 in D11 in 1.55 mg TPH-DRO corresponding to 9.9 mg/mL TPH-ORO. **C2**: D13 in 31.1 mg TPH-DRO corresponding to 198 mg/mL TPH-ORO.. (): Week 2, (): Week 4, (): Week 6 and (): Week 14.

The FTIR analyses of the cultural components of pure cultures of D11, D12, and D13 at 1.55 mg/mL and 31.1 mg/mL TPH-DRO (9.9 mg/mL and 198 mg/mL TPH-ORR, respectively) after 14 weeks incubation are shown in Fig. 6. The spectra registered at the various hydrocarbons concentrations show similar appearance, with some changes in the ranges 1000−1500 cm^−1^ and 1700−3000 cm^−1^. FTIR spectra show two main peaks, one at 3358 cm^−1^ and the other at 1640 cm^−1^. This indicates that there are presence of −OH group. The peak corresponding to 1640 cm^−1^ demonstrates that the cultures contain carboxylic acids. The overtones and combination of bands peaked at 2172 cm^−1^ indicate that some alkanes are still present in the cultures.

**Fig. 7:**
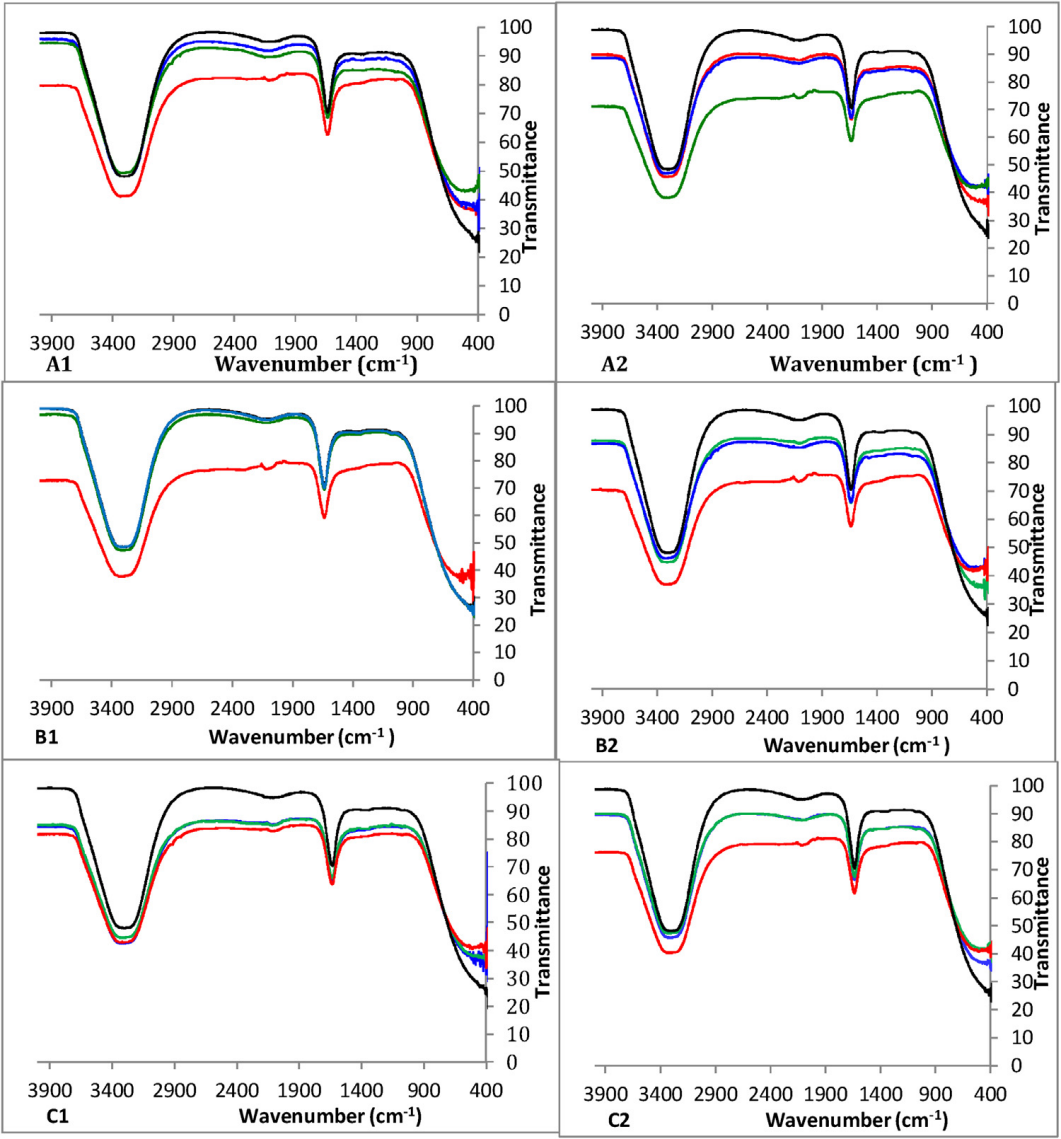
FTIR analyses performed in the cultures performed with: A1: D11/D12 in 1.55 mg TPH-DRO corresponding to 9.9 mg/mL TPH-ORO. A2: D11/D12 in 31.1 mg TPH-DRO corresponding to 198 mg/mL TPH-ORO. B1: D11/D13 in 1.55 mg TPH-DRO corresponding to 9.9 mg/mL TPH-ORO. B2: D11/D13 in 31.1 mg TPH-DRO corresponding to 198 mg/mL TPH-ORO. C1: D12/D13 in 1.55 mg TPH-DRO corresponding to 9.9 mg/mL TPH-ORO. C2: D12/D13 in 31.1 mg TPH-DRO corresponding to 198 mg/mL TPH-ORO.. (): Week 2, (): Week 4, (): Week 6 and (): Week 14.

In combined cultures (Fig. 7 A and B), the FTIR spectra of D11/D12 show D12 caused an increase in the magnitude of infrared radiation. At time 0–2 weeks, high absorbance of peaks in hydrocarbons reaching 31.1 mg/mL TPH-DRO with 198 mg/mL TPH-ORO. However, with 311 mg/mL TPH-DRO and 1980 mg/mL TPH-ORO, the changes in the spectra are less clear. The sharp peak located at around 1640 cm^−1^ proves the presence of stretching –C=C-. Small changes occur at a peak of 2190 cm^−1^, representing alkyne (C≡C) stretching. All these changes show that that D11/D12 developed together an adaptation requiring a longer time at the high TPH concentrations, but start to cause changes in the overall composition after 14 weeks, even at the high TPH concentration.

In the co-cultures performed with D11/D13, changes in the FTIR spectra were similar to the case of D11/D12 cultures (Fig. 7 C and D). However, at the 100 times concentrated hydrocarbons (with 311 mg/mL TPH-DRO and 1980 mg/mL TPH-ORO) there was some overtone peaks vibration and combination bands at 2138 cm^−1^, at week 4, while at week 14 these overtones nearly disappeared due to longer incubation with bacteria.

FTIR spectra of D12/D13 cultures (Fig. 7 E and F) show that the culture absorbed a high magnitude of infrared radiation at 31.1 mg/mL TPH-DRO and 198 mg/mL TPH-ORO. An overtone and combination of bands at 1655 cm^−1^ are clear, which indicates residual alkenes C=C stretching. With high TPH concentrations (311 mg/mL TPH-DRO and 1980 mg/mL TPH-ORO), peaks started to shift to higher transmittance which as affected by bacterial activity.

**Fig. 8:**
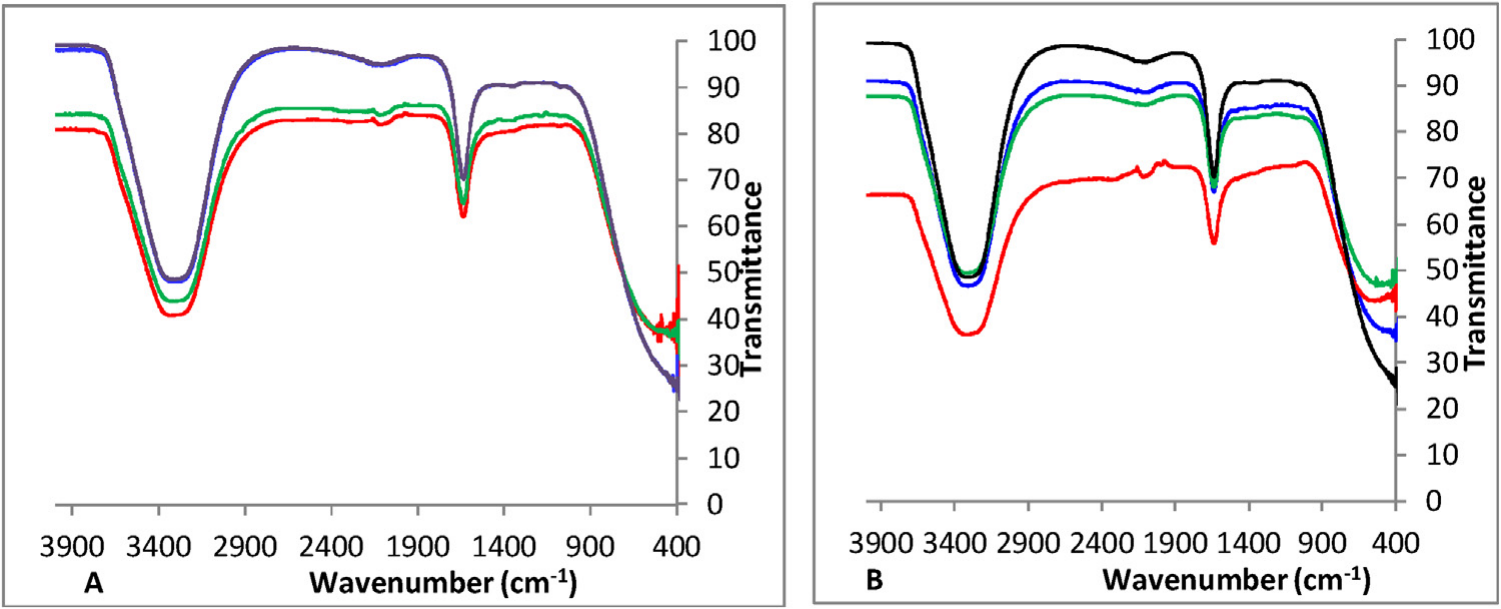
FTIR analyses performed in the cultures performed with : **A** : D11/D12/D3 in 0.311 mg TPH-DRO corresponding to 198 mg/mL TPH-ORO. **B**: D11/D12/D3 in 1.55 mg TPH-DRO corresponding to 9.9 mg/mL TPH-ORO. (): Week 2, (): Week 4, (): Week 6 and (): Week 14.

With the combination of the three strains D11/D12/D13 (Fig. 8), FTIR spectra show that at low hydrocarbons concentration (Fig. 8. A), there is a clear sharp peak during the first two weeks at around 1635 cm^−1^, which indicates -C=C-. At the100 times hydrocarbons concentration (31.1 mg/mL TPH-DRO and 198 mg/mL TPH-ORO), a new small peak appears at 2149 cm^−1^ (alkyne (C≡C) stretching). It found that aliphatic hydrocarbon C–H bonds, in contrary the C≡C hydrocarbons bonds (due to bonded OH) are largely degraded during the treatment.

## 3 Conclusion

The oily-wastes dumping site in the area of Dukhan, Qatar, was considered as a model of soils subjected to all types of weathering processes. Initially, the soils contained 3.11 mg/g TPH-DRO and 19.8 mg/g TPH-ORO. C_29_-C_35_ hydrocarbons represent 6.4 folds of the DRO fraction (C_10_-C_28_ range). The three endogenous bacteria (*Bacillus sorensis* D11*, Bacillus cereus* D12, *and Pseudomonas stutzeri* D13) were used for the self-purification of the soil. The processes of biostimulation/seeding using one or another of the indigenous bacteria lead to low performance of TPH removal. The symbiosis between the three strains dominating the soil, was studied in Mineral Salts Medium supplemented with total petroleum hydrocarbons (TPH) extracted from the weathered soil. Indeed, the mixed bacterial strains could reduce the lag phase during the first weeks of incubation. D11 (*Bacillus sorensis*) showed the highest growth of all the three strains at low TPH concentrations. D13 (*Pseudomonas stutzeri*) experienced the least rate of growth. When D11 and D13 were incubated together, D13 exhibited the highest growth in week 6 while D11 peaked in week 4. D11 was shown more efficient in such activity, but also more sensitive to exhaustion of substrates or the toxic effect of intermediates or end-products. The combination of 2 or 3 strains, allowed the decrease of the lag phase. D11 is more active on the TPH-DRO range, leading to an increase in the ratio ORO/DRO to 7.5 compared to the initial one of 6.4. D12 was less performant to remove TPH than D11. It seems that the co-metabolic activities of D11 and D12 would be interesting. D13 which was more active on C_29_-C_35_ molecules than C_10_-C_28_. It can only improve the TPH removal in the combinations D11/D12 or D11/D12/D13. The symbiosis of D13 to D11 and D13 seems to be closer to a commensal one more than a mutualistic one.

It is obviously clear that the behavior of the separate isolates or their combinations is different at each hydrocarbons concentration. D11 and D12 are the most sensitive to toxicity. However, in the combinations of strains, D11 showed a better tolerance to TPH toxicity. The same conclusion can be drawn for D12 and D13. The growth and use of TPH as carbon and energy sources of each separate or combined strains depends on the concentration of each component in the TPH extract. Symbiosis between the bacterial communities seems also to be affected by the concentration of TPH.

With the combination of the three strains D11/D12/D13, FTIR spectra showed that at low hydrocarbons concentration), there is a clear sharp peak during the first two weeks at around 1635 cm^−1^, which indicates -C=C-. At the100 times hydrocarbons concentration (31.1 mg/mL TPH-DRO and 198 mg/mL TPH-ORO), a new small peak appears at 2149 cm^−1^ (alkyne (C≡C) stretching).

## Funding

The publication of this article was funded by the Qatar National Library.

## Declaration of Competing Interest

The authors report no declarations of interest.
